# Supplementary material for: Electromyographic analysis of the stomatognathic system of children with Molar-incisor hypomineralization
Source: PLoS One. 2023 Feb 24;18(2):e0277030. doi: 10.1371/journal.pone.0277030 (PMC9955966; doi:10.1371/journal.pone.0277030)
Supplement: S1 File — (PDF) [file pone.0277030.s002.pdf]

Teste-T

Observações

Missing value treatment	Definition of missing	User-defined missing values are treated as missing.
	Casos utilizados	The statistics for each analysis are based on cases without missing or out-of-amplitude data for any variable in the analysis.
Syntax		T-TEST GROUPS=grupo(1 2)<br>/MISSING=ANALYSIS<br>/VARIABLES=idade IMC tscamd tscame tscam16 tscam26 tscam36 tscam46 fmd fme iopibucinador iopilingua iopilabio mmmmd mmmme mmmtd mmmte mmmod mmmoe mmms mbismd mbisme mbistd mbiste mbisod mbisoe mbiss rmd rme rtd rte rod roe rsupra ldmd ldme ldtd ldte ldod ldoe ldsupra lemd leme letd lete leod leoe lesupra pmd pme ptd pte pod poe psupra<br>/CRITERIA=CI(.95).

Data

Grupo 1 =MIH e Grupo 2 =Control		N	Media	SD	Standard average error
Age	1,00	36	8,3056	1,65304	,27551
	2,00	36	8,5556	,84327	,14055

BMI	1,00		36	19,9639	5,65407	,94235
	2,00		36	18,8119	4,52310	,75385
Chewing MMs masseter right	1,00		36	1,0628	,99793	,16632
	2,00		36	,9278	,96129	,16022
Chewing MMs left masseter	1,00		36	1,0942	,93623	,15604
	2,00		36	,9678	,90000	,15000
Chewing mms temporal right	1,00		36	,9700	1,05435	,17573
	2,00		36	,6950	,28489	,04748
Chewing left temporal MMs	1,00		36	,9467	,91034	,15172
	2,00		36	,5544	,25193	,04199
Chewing Bis masseter right	1,00		36	,8981	,78083	,13014
	2,00		36	,8772	,50749	,08458
Chewing Bis masseter left	1,00		36	1,1436	1,29375	,21563
	2,00		36	,8656	,89240	,14873
Right Temporal Bis Mastication	1,00		36	1,1942	1,34118	,22353
	2,00		36	,6081	,30980	,05163
Left Temporal Bis Mastication	1,00		36	,9058	,79432	,13239
	2,00		36	,4939	,21054	,03509
	2,00		36	1,6700	,66298	,11050
Right masseter rest	1,00		36	,1192	,17796	,02966
	2,00		36	,1178	,12538	,02090
left masseter rest	1,00		36	,1356	,14657	,02443
	2,00		36	,0997	,10027	,01671
Right temporal rest	1,00		36	,5919	,40082	,06680
	2,00		36	,1369	,10757	,01793
Left temporal rest	1,00		36	,2458	,35485	,05914
	2,00		36	,1119	,08046	,01341
Right side masseter right	1,00		36	1,6597	2,75358	,45893
	2,00		36	,2606	,48966	,08161
Right side left masseter	1,00		36	,3089	,40314	,06719
	2,00		36	,5283	,93395	,15566
Laterality Right temporal right	1,00		36	,3094	,49880	,08313
	2,00		36	,2814	,44987	,07498
Left temporal right laterality	1,00		36	,2100	,23515	,03919

	2,00		36	,1061	,06189	,01031
Left side masseter right	1,00		36	1,6272	3,54032	,59005
	2,00		36	,3178	,36837	,06140
Left side left masseter left	1,00		36	,2739	,30707	,05118
	2,00		36	,1972	,17164	,02861
Laterality Left temporal right	1,00		36	,2647	,36073	,06012
	2,00		36	,1414	,14389	,02398
Left left laterality left temporal	1,00		36	,2922	,29676	,04946
	2,00		36	,1844	,09449	,01575
Protrusion masseter right	1,00		36	,7831	1,45188	,24198
	2,00		36	,6381	,97082	,16180
Protrusion left masseter	1,00		36	,7636	1,33516	,22253
	2,00		36	,5664	,83606	,13934
Right temporal protrusion	1,00		36	,3639	,44414	,07402
	2,00		36	,1858	,18320	,03053
Left temporal protrusion	1,00		36	,2789	,39171	,06528
	2,00		36	,1606	,20046	,03341

Independent sample testing

		Levene test for equality of variances			
		F	Sig.	t	df
age	Equal variances assumed	20,005	,000	-,808	70
	Equal variances not assumed			-,808	52,061
BMI	Equal variances assumed	2,522	,117	,955	70
	Equal variances not assumed			,955	66,781
Chewing MMs masseter right	Equal variances assumed	1,791	,185	,585	70

	Equal variances not assumed			,585	69,902
Chewing MMs left masseter	Equal variances assumed	,374	,543	,584	70
	Equal variances not assumed			,584	69,891
Chewing mms temporal right	Equal variances assumed	8,700	,004	1,511	70
	Equal variances not assumed			1,511	40,084
Chewing left temporal MMs	Equal variances assumed	10,005	,002	2,491	70
	Equal variances not assumed			2,491	40,330
Chewing Bis masseter right	Equal variances assumed	6,998	,010	,134	70
	Equal variances not assumed			,134	60,092
Chewing Bis masseter left	Equal variances assumed	1,283	,261	1,061	70
	Equal variances not assumed			1,061	62,157
Right Temporal Bis Mastication	Equal variances assumed	10,411	,002	2,555	70
	Equal variances not assumed			2,555	38,724
Mastigação Bis temporal esquerdo	Equal variances assumed	12,075	,001	3,008	70
	Equal variances not assumed			3,008	39,894
Right masseter home	Equal variances assumed	,324	,571	,038	70
	Equal variances not assumed			,038	62,878
Rest left masseter	Equal variances assumed	2,165	,146	1,211	70

	Equal variances not assumed			1,211	61,875
Right temporal rest	Equal variances assumed	41,040	,000	6,578	70
	Equal variances not assumed			6,578	40,015
Left temporal rest	Equal variances assumed	7,707	,007	2,208	70
	Equal variances not assumed			2,208	38,589
Right side masseter right	Equal variances assumed	14,355	,000	3,002	70
	Equal variances not assumed			3,002	37,211
Right side left masseter	Equal variances assumed	7,192	,009	-1,294	70
	Equal variances not assumed			-1,294	47,605
Laterality Right temporal right	Equal variances assumed	,007	,932	,251	70
	Equal variances not assumed			,251	69,267
Left temporal right laterality	Equal variances assumed	11,397	,001	2,563	70
	Equal variances not assumed			2,563	39,825
Left side masseter right	Equal variances assumed	10,198	,002	2,207	70
	Equal variances not assumed			2,207	35,758
Left side left masseter left	Equal variances assumed	2,663	,107	1,308	70
	Equal variances not assumed			1,308	54,926
Laterality Left temporal right	Equal variances assumed	7,585	,007	1,905	70

	Equal variances not assumed			1,905	45,862
Left left laterality left temporal	Equal variances assumed	9,067	,004	2,076	70
	Equal variances not assumed			2,076	42,024
Protrusion masseter right	Equal variances assumed	,206	,651	,498	70
	Equal variances not assumed			,498	61,083
Protrusion left masseter	Equal variances assumed	,455	,502	,751	70
	Equal variances not assumed			,751	58,790
Right temporal protrusion	Equal variances assumed	8,345	,005	2,224	70
	Equal variances not assumed			2,224	46,575
Left temporal protrusion	Equal variances assumed	5,278	,025	1,614	70
	Equal variances not assumed			1,614	52,156

t-test for Average Equality				
Sig. (2 ends)	Average difference	Standard difference error	95% Difference confidence interval	
			Inferior	Superior
,422	-,25000	,30928	-,86685	,36685
,423	-,25000	,30928	-,87061	,37061
,343	1,15194	1,20677	-1,25489	3,55878
,343	1,15194	1,20677	-1,25693	3,56082
,561	,13500	,23094	-,32559	,59559

	,561	,13500	,23094	-,32560	,59560
	,561	,12639	,21644	-,30530	,55807
	,561	,12639	,21644	-,30531	,55809
	,135	,27500	,18203	-,08804	,63804
	,139	,27500	,18203	-,09287	,64287
	,015	,39222	,15743	,07825	,70620
	,017	,39222	,15743	,07413	,71031
	,894	,02083	,15521	-,28872	,33039
	,894	,02083	,15521	-,28962	,33129
	,292	,27806	,26195	-,24438	,80049
	,293	,27806	,26195	-,24554	,80165
	,013	,58611	,22942	,12855	1,04367
	,015	,58611	,22942	,12197	1,05025
	,004	,41194	,13696	,13879	,68510
	,005	,41194	,13696	,13512	,68877
	,970	,00139	,03628	-,07097	,07375
	,970	,00139	,03628	-,07112	,07390
	,230	,03583	,02960	-,02320	,09486

,231	,03583	,02960	-,02333	,09500
,000	,45500	,06917	,31705	,59295
,000	,45500	,06917	,31521	,59479
,031	,13389	,06064	,01294	,25484
,033	,13389	,06064	,01119	,25659
,004	1,39917	,46613	,46950	2,32883
,005	1,39917	,46613	,45488	2,34346
,200	-,21944	,16954	-,55758	,11869
,202	-,21944	,16954	-,56040	,12151
,803	,02806	,11195	-,19522	,25133
,803	,02806	,11195	-,19526	,25137
,013	,10389	,04053	,02306	,18472
,014	,10389	,04053	,02197	,18581
,031	1,30944	,59324	,12627	2,49262
,034	1,30944	,59324	,10602	2,51287
,195	,07667	,05863	-,04027	,19360
,196	,07667	,05863	-,04084	,19417
,061	,12333	,06473	-,00576	,25243

,063	,12333	,06473	-,00697	,25364
,042	,10778	,05191	,00425	,21130
,044	,10778	,05191	,00303	,21253
,620	,14500	,29109	-,43557	,72557
,620	,14500	,29109	-,43706	,72706
,455	,19722	,26255	-,32643	,72087
,456	,19722	,26255	-,32819	,72263
,029	,17806	,08007	,01835	,33776
,031	,17806	,08007	,01693	,33918
,111	,11833	,07334	-,02793	,26460
,113	,11833	,07334	-,02882	,26548
