# Supplementary material for: Electromyographic analysis of the stomatognathic system of children with Molar-incisor hypomineralization
Source: PLoS One. 2023 Feb 24;18(2):e0277030. doi: 10.1371/journal.pone.0277030 (PMC9955966; doi:10.1371/journal.pone.0277030)
Supplement: Attachment — Submitted filename: 10.2022 - Response to Reviewers (1).docx [file pone.0277030.s003.docx]

Ribeirão Preto, October 13^th^, 2022

Editor-in-Chief

Emily Chenette

PlosOne

**Manuscript** **PONE-D-22-20949:** Electromyographic Analysis of the Stomatognathic System of Children with Molar-Incisor Hypomineralization

Dear Professor Emily Chenette

Thank you so much for allowing us to review the manuscript **PONE-D-22-20949**. In the following, we respond to each of the referee's points and explain the resulting changes in the manuscript. The changes incorporated in the revised manuscript are in bold. The revised manuscript in its submitted form has been read and approved by all authors.

Sincerely yours,

Professor Carvalho.

**Manuscript PONE-D-22-20949:** Electromyographic Analysis of the Stomatognathic System of Children with Molar-Incisor Hypomineralization

**Dear Dr. Carvalho,**

We've checked your submission and before we can proceed, we need you to address the following issues:

1. Re: Table

Please include a copy of Table 10 which you refer to in your text on page 4.

**Answer: The tables were corrected in the text.**

2. Can you please upload an additional copy of your revised manuscript that does not contain any tracked changes or highlighting as your main article file. This will be used in the production process if your manuscript is accepted. Please amend the file type for the file showing your changes to Revised Manuscript w/tracked changes. Please follow this link for more information: <http://blogs.PLOS.org/everyone/2011/05/10/how-to-submit-your-revised-manuscript/>

**Answer: The corrections were made in the text.**
